# Variability in Resting State Network and Functional Network Connectivity Associated With Schizophrenia Genetic Risk: A Pilot Study

**DOI:** 10.3389/fnins.2018.00114

**Published:** 2018-03-01

**Authors:** Jiayu Chen, Barnaly Rashid, Qingbao Yu, Jingyu Liu, Dongdong Lin, Yuhui Du, Jing Sui, Vince D. Calhoun

**Affiliations:** ^1^Mind Research Network, Albuquerque, NM, United States; ^2^Harvard Medical School, Harvard University, Boston, MA, United States; ^3^Department of Electrical Engineering, University of New Mexico, Albuquerque, NM, United States; ^4^School of Computer & Information Technology, Shanxi University, Taiyuan, China; ^5^Brainnetome Center and National Laboratory of Pattern Recognition, Institute of Automation, Chinese Academy of Sciences, Beijing, China; ^6^Departments of Neurosciences and Psychiatry, University of New Mexico School of Medicine, Albuquerque, NM, United States

**Keywords:** variability, resting state network, functional network connectivity, schizophrenia, PGC, parallel ICA

## Abstract

Imaging genetics posits a valuable strategy for elucidating genetic influences on brain abnormalities in psychiatric disorders. However, association analysis between 2D genetic data (subject × genetic variable) and 3D first-level functional magnetic resonance imaging (fMRI) data (subject × voxel × time) has been challenging given the asymmetry in data dimension. A summary feature needs to be derived for the imaging modality to compute inter-modality association at subject level. In this work, we propose to use variability in resting state networks (RSNs) and functional network connectivity (FNC) as potential features for purpose of association analysis. We conducted a pilot study to investigate the proposed features in a dataset of 171 healthy controls and 134 patients with schizophrenia (SZ). We computed variability in RSN and FNC in a group independent component analysis framework and tested three types of variability metrics, namely Euclidean distance, Pearson correlation and Kullback-Leibler (KL) divergence. Euclidean distance and Pearson correlation metrics more effectively discriminated controls from patients than KL divergence. The group differences observed with variability in RSN and FNC were highly consistent, indicating patients presenting increased deviation from the cohort-common pattern of RSN and FNC than controls. The variability in RSN and FNC showed significant associations with network global efficiency, the more the deviation, the lower the efficiency. Furthermore, the RSN and FNC variability were found to associate with individual SZ risk SNPs as well as cumulative polygenic risk score for SZ. Collectively the current findings provide preliminary evidence for variability in RSN and FNC being promising imaging features that may find applications as biomarkers and in imaging genetic association analysis.

## Introduction

Most psychiatric disorders have been characterized to present moderate to high heritability in family and twin studies (Kendler and Eaves, 2005). In the past decade, advancement in high-throughput genetic profiling techniques has enabled further characterization of the underlying genetic structure. Particularly for the five major psychiatric disorders, schizophrenia (SZ), bipolar disorder (BD), major depression, autism and attention deficit/hyper activity (ADHD), large genome wide association studies (GWAS) lend support for their polygenic nature, where single nucleotide polymorphisms (SNPs) were estimated to explain 17–29% of the variance in liability (Lee et al., 2013). Among the five disorders, the most well-characterized is schizophrenia, for which 108 genome-wide significant risk loci have been identified in a large GWAS of 36,989 cases and 113,075 controls (Ripke et al., 2014). Meanwhile, sample size is continuing to increase and has started to yield potential risk loci for other disorders (Sklar, 2012; Sullivan et al., 2013; Anney et al., 2017).

From the neurobiological perspective, patients with psychiatric disorders present brain structural and functional abnormalities, which may underlie the clinical manifestations. Characteristic abnormalities include extensive gray matter reductions compared with healthy controls and aberrant regional activations during various cognitive tasks (Manoach et al., 2000; Monks et al., 2004; Harris et al., 2006; Green et al., 2007; Ivleva et al., 2012). Meanwhile, there has been growing interest in resting state functional network analysis, where disrupted network coherence (He et al., 2007; Yu et al., 2013; Xu et al., 2015) and inter-network connectivity have been reported in several psychiatric disorders, including SZ (Calhoun et al., 2011; Bassett et al., 2012; Meda et al., 2012; Manoliu et al., 2013; Rashid et al., 2014) and BD (Calhoun et al., 2011; Yu et al., 2011; Meda et al., 2012; Rashid et al., 2014). Notably, resting state networks are suggested to be heritable (Glahn et al., 2010; Fu et al., 2015). Glahn et al. has reported a heritability of 0.42 for the default-mode network connectivity. In line with this, aberrant resting state functions have been noted in people at high risk of developing psychiatric disorders (Meda et al., 2016).

Thus there is a pressing need to characterize genetic underpinnings of brain abnormalities in psychiatric disorders which helps elucidate the biological mechanisms and inspire treatment therapies. A direct test on individual mutations' associations with individual brain phenotypes is straightforward, and has identified genetic loci related to reduced volumes in putamen and hippocampus (Hibar et al., 2015, 2017). However, this type of analysis in general suffers from insufficient power due to the moderate effect sizes of individual genetic variants. In contrast, multivariate multimodal techniques boost statistical power by mining genetic/imaging data to capture the interactive or integrated effect of multiple genetic variants/brain regions and assess the covariation or association of multiple data modalities, which is also considered as a type of fusion (Chen et al., 2013b; Liu and Calhoun, 2014; Vergara et al., 2014; Calhoun and Sui, 2016). Parallel independent component analysis (pICA) is a technique designed for this purpose, building upon ICA to conduct separate multivariate analysis in two data modalities and then optimizing the inter-modality association (Liu and Calhoun, 2014). It has found wide applications in integrating genetic and neuroimaging data including gray matter density, gray matter volume, and task-related activation (Meda et al., 2014; Pearlson et al., 2015).

While parallel ICA is designed for imaging genetic association analysis, its application is restricted to second-level functional magnetic resonance imaging (fMRI) data (i.e., either a time dimension or a spatial dimension, but not both; Liu et al., 2009; Meda et al., 2014). It would be desirable to extend the pICA approach to link genetics to first-level functional data for direct association optimization, which might better reveal genetic influence on coherent functional networks. To achieve data fusion between 2D genetic data (e.g., subject × SNP) and 3D neuroimaging data (e.g., subject × voxel × time), a summary feature needs to be derived for the imaging modality to associate with the genetic modality at subject level. Here we propose to utilize two features including variability in resting state network (RSN) and variability in functional network connectivity (FNC). Variability in RSN has been explored by Damoiseaux et al. (2006) in the context of cross-session reliability. Another study compared the overall inter-subject variability as measured with pairwise correlation in early-blinded subjects with that in sighted subjects to infer differences in cortical reorganization (Boldt et al., 2014). A more relevant work by Finn et al. has demonstrated that the functional connectivity profile of a specific subject obtained from one session is more similar to his/her own functional connectivity profile than others' obtained from a different session of the same condition, lending support for substantial and reproducible individual variability in connectivity (Finn et al., 2015). However, neither of these studies sought to quantify the individual variability at network or overall connectivity level, which may serve as potential neuroimaging biomarkers.

In the current work, we demonstrate a framework to estimate the variability in subject-specific RSN and FNC based on spatial group ICA. Theoretically, group-level components capture spatial coactivation patterns in brain shared across all the subjects, each component is an independent brain network. In this sense, a group-level component can be interpreted as a cohort-common pattern presenting a functional network template conserved in all the subjects. Consequently, it is worthwhile to investigate how much the RSN (i.e., component in resting state fMRI) and resulting FNC (i.e., cross correlation among components' time courses) deviate from the cohort-common pattern and whether this deviation may serve as a biomarker and be regulated by genetics. Our results suggest that variability in RSN and FNC consistently discriminate controls from patients with SZ and show preliminary SNP associations, lending support for it being a promising feature in imaging genetic association analysis.

## Materials and methods

### Participants

We tested the proposed framework in a total of 305 subjects with good quality SNP and resting fMRI data aggregated from COBRE and FBIRN studies. Details regarding recruitment and data collection can be found in our previous publications (Damaraju et al., 2014; Yu et al., 2015; Aine et al., 2017; Chen et al., in press). The cohort consisted of 171 controls (123 males, 48 females; mean age 37.81) and 134 patients with SZ (115 males, 19 females; mean age 37.76). Informed consent was obtained from each participant prior to scanning in accordance with the Internal Review Boards of corresponding institutions. For the FBIRN study, 162 volumes, and for the COBRE study, 149 volumes of resting state scans were collected on 3-Tesla scanners with a TR of 2 s.

### fMRI data preprocessing

Data processing was performed using a combination of toolboxes including AFNI (http://afni.nimh.nih.gov/afni), SPM (http://www.fil.ion.ucl.ac.uk/spm/software), GIFT (http://mialab.mrn.org/software/gift), and custom code written in Matlab. Imaging data were preprocessed using an automated SPM8-based preprocessing pipeline. Images were realigned using INRIalign (http://www-sop.inria.fr/epidaure/Collaborations/IRMf/INRIAlign.html) and slice-time correction was applied using the middle slice as the reference frame. Data were then spatially normalized to standard MNI space (http://www.mni.mcgill.ca/) and resampled to 3 × 3 × 3 mm voxels using the nonlinear registration implemented in the SPM toolbox. Finally, data were smoothed using 6 mm FWHM Gaussian kernel.

After initial standard preprocessing, the imaging data was decomposed into functionally homogeneous cortical and subcortical regions exhibiting temporally coherent activity using a high model order (75) group-level spatial ICA. Group ICA is an extension of ICA model that can be applied to group data for estimation of a set of common sources for all subjects (Calhoun et al., 2001; Calhoun and Adali, 2012).

### SNP data preprocessing

The genotyping and genetic quality control procedures were same as described in our previous work (Chen et al., in press), which is briefly summarized here. DNA was extracted from blood or saliva samples. Illumina Human Omni1-Quad, Illumina Human Omni5, and Illumina Infinium MEGAEX + Psych were used for genotyping. No significant difference was noted in genotyping call rates between blood and saliva samples. A standard quality control (QC) (Chen et al., 2013b) was performed using PLINK (Purcell et al., 2007). Then imputation was conducted with SHAPEIT for pre-phasing (Delaneau et al., 2012), IMPUTE2 for imputation (Marchini and Howie, 2010), and the 1,000 Genomes data as the reference panel (Altshuler et al., 2012). Only markers with high imputation qualities (INFO score > 0.95) were retained. Finally linkage disequilibrium (LD) pruning (*r*^2^ > 0.9) was applied to yield 977,242 SNPs for which population structure was corrected using principal component analysis (Price et al., 2006).

### Variability in RSN and FNC

**Figure 1:**
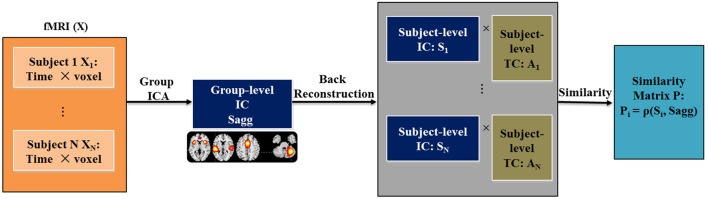
Graphic presentation of the proposed similarity matrix estimation.

For each spatial brain component and each subject, we computed the Euclidean distance, Pearson correlation and Kullback-Leibler (KL) divergence between the subject-level component and the group-level component to measure variability in RSN. Figure 1 presents a flowchart of the proposed framework for computing variability. The resting fMRI data is decomposed with spatial group ICA, before which a cohort-common group mask is first generated using the following method: (1) For each subject, we obtain the subject-level mask by locating the voxels that have activations greater than the mean activation across all the voxels; (2) We obtain the group mask as the intersection of all the subject-level masks. Only voxels in the group mask will be included in the subsequent group ICA analysis. The masked 2D subject-level data are concatenated along the time dimension for N subjects. Group-level components are estimated from the aggregated data, denoted as Sagg. Following the component estimation, subject-level components and time courses (S_i_ and A_i_ for *i*th subject) are recovered from Sagg through back reconstruction. Then for each subject, variability metric is computed between S_i_ and Sagg to quantify the variability of his/her function networks from the cohort-common pattern. The resulting variability matrix is denoted as P. For exploratory purpose, we tested three types of commonly used metric of difference, including Euclidean distance (ρ_*ED*_), Pearson correlation (ρ_*PC*_), and KL divergence (ρ_*KL*_), which enabled us to examine both linear and nonlinear relationships of similarity between the two patterns, as listed in Equations (1–3). The subscript i and j represent the subject and component index, respectively. K denotes the number of voxels and M denotes the number of bins for the histogram Q. Note that the subject-level variability in RSN is computed between subject-level component and group-level component that both span the whole group mask. This avoids underestimating the variability for someone who has an RSN pattern greatly different from the cohort-common pattern. In the current work, 75 components were extracted from group ICA following our previous work (Yu et al., 2011). For the computation of KL divergence, the number of bins was set to 100. The resulting variability features were evaluated for case vs. control differences while controlling for age, sex and dummy-coded site covariates, considering that various scanning protocols were used in data collection.

We also evaluated the variability in FNC as a potential imaging feature. The connectivity matrix C was constructed as the cross-correlations among components' time courses, as shown in Equation (4) where i denotes subject index, j1 and j2 denote component indices. Then variability metric can be computed between the individual connectivity matrix C_i_ and the mean connectivity across all the subjects ($\bar{C}$). Equation (5) shows an example for the computation of Euclidean distance, where D stands for the number of connections. Specifically in the current work, 50 of 75 components were selected for FNC analysis following a common practice that (1) the components were characterized as not related to physiological signal, movement, or imaging artifacts; and (2) the components fell into the major cortical networks commonly used to construct FNC (Yu et al., 2011, 2015; Allen et al., 2014). The time courses of these 50 components were detrended, motion corrected, despiked and bandpass filtered at 0.01–0.08 Hz. An FNC matrix was constructed for each subject based on the correlations among time courses of these 50 components. Then the variability feature was computed based on normalized FNC (z-score) and evaluated for case vs. control difference while controlling for age, sex and dummy-coded site covariates. Considering that there were more controls than patients in this cohort, which might bias the results given that the mean FNC was employed as the reference pattern in this preliminary analysis, we repeated the above analysis using a balanced subcohort of 134 patients and 134 controls.

(1) $$\begin{array}{l} {\text{P}}_{i,j}\text{=}\rho_{ED}\left(S_{i,j},S_{agg,j}\right)\\ \;=\sqrt{\sum_{k=1}^{K}{\left({\left[S_{i,j}\right]}_{k}-{\left[S_{agg,j}\right]}_{k}\right)}^{2}} \end{array}$$

(2) $$\begin{array}{l} {\text{P}}_{i,j}\text{=}\rho_{PC}\left(S_{i,j},S_{agg,j}\right)\\ \;=\frac{\sum_{k=1}^{K}\left({\left[S_{i,j}\right]}_{k}-\bar{S_{i,j}}\right)\left({\left[S_{agg,j}\right]}_{k}-\bar{S_{agg,j}}\right)}{\sqrt{\sum_{k=1}^{K}{\left({\left[S_{i,j}\right]}_{k}-\bar{S_{i,j}}\right)}^{2}}\sqrt{\sum_{k=1}^{K}{\left({\left[S_{agg,j}\right]}_{k}-\bar{S_{agg,j}}\right)}^{2}}} \end{array}$$

(3) $$\begin{array}{l} {\text{P}}_{i,j}\text{=}\rho_{KL}\left(Q\left(S_{i,j}\right)||Q\left(S_{agg,j}\right)\right)\\ \;=\sum_{m=1}^{M}Q{\left(S_{i,j}\right)}_{m}\log\frac{Q{\left(S_{i,j}\right)}_{m}}{Q{\left(S_{agg,j}\right)}_{m}} \end{array}$$

(4) $${\left[C_{i}\right]}_{j1,j2}=\frac{cov\left(A_{i,j1},A_{i,j2}\right)}{\sigma \left(A_{i,j1}\right)\sigma \left(A_{i,j2}\right)}$$

(5) $${\text{P}}_{\text{i}}\text{=}\rho_{ED}\left(C_{i},\bar{C}\right)\text{=}\sqrt{\sum_{d=1}^{D}{\left({\left[C_{i}\right]}_{d}-{\left[\bar{C}\right]}_{d}\right)}^{2}}$$

### Variability vs. global efficiency

For a physiological interpretation, we assessed the relationship between proposed features of variability in RSN and FNC with FNC global efficiency. Global efficiency is known as measuring the overall efficiency of parallel information transfer in the network, which is defined as the inverse of the harmonic mean of the minimum path length between each pair of nodes (Latora and Marchiori, 2001). Specifically in this work, the global efficiency was computed using weighted and undirected connectivity matrix following (Rubinov and Sporns, 2010), where absolute values of time course correlations were used to capture the connection strength. Finally the average of the nodal level global efficiencies was computed to reflect the holistic network efficiency. This global efficiency measure was then evaluated for group difference as well as correlations with variability in RSN and FNC.

### Genetic association with variability in RSN and FNC

The group-discriminating variability features were further investigated for potential genetic associations. Two sets of SNPs were selected for investigation, including: (a) a set of 5,907 SZ risk SNPs that showed a group discrimination *p*-value < 1 × 10^−4^ in the largest psychiatric genomic consortium (PGC) SZ GWAS, denoted as PGC set; and (b) a set of 418 SNPs residing in the *BDNF* and *CREB* family genes known to be involved in schizophrenia and neural development (Bramham and Messaoudi, 2005; Carlezon et al., 2005), denoted as CREB-BDNF set. For each SNP set, we first evaluated the associations between the identified group-discriminating RSN/FNC variability features and the individual SNPs using regression, where the variability in RSN/FNC was modeled as a function of a single SNP. Bonferroni correction was used to guard against false positives. In addition, we computed the polygenic risk score (PRS) for SZ of the whole set of SNPs, which was a linearly weighted sum of the genotype profiles with weights derived from the odds ratios of the PGC SZ GWAS (Purcell et al., 2009; Ripke et al., 2014). The group-discriminating variability features were then assessed for associations with PRS for SZ using regression where the variability feature was modeled as a function of PRS for SZ.

### Data and code availability

Data analyzed in this study were aggregated from COBRE and FBIRN studies. The COBRE data have been deposited through COINS (https://coins.mrn.org). Availability of the FBIRN data is upon request from the principal investigator: Dr. Steven G. Potkin. We plan to release software implementing the approach within the Fusion ICA Toolbox (FIT: http://mialab.mrn.org/software/fit).

## Results

### Variability in RSN

**Figure 2:**
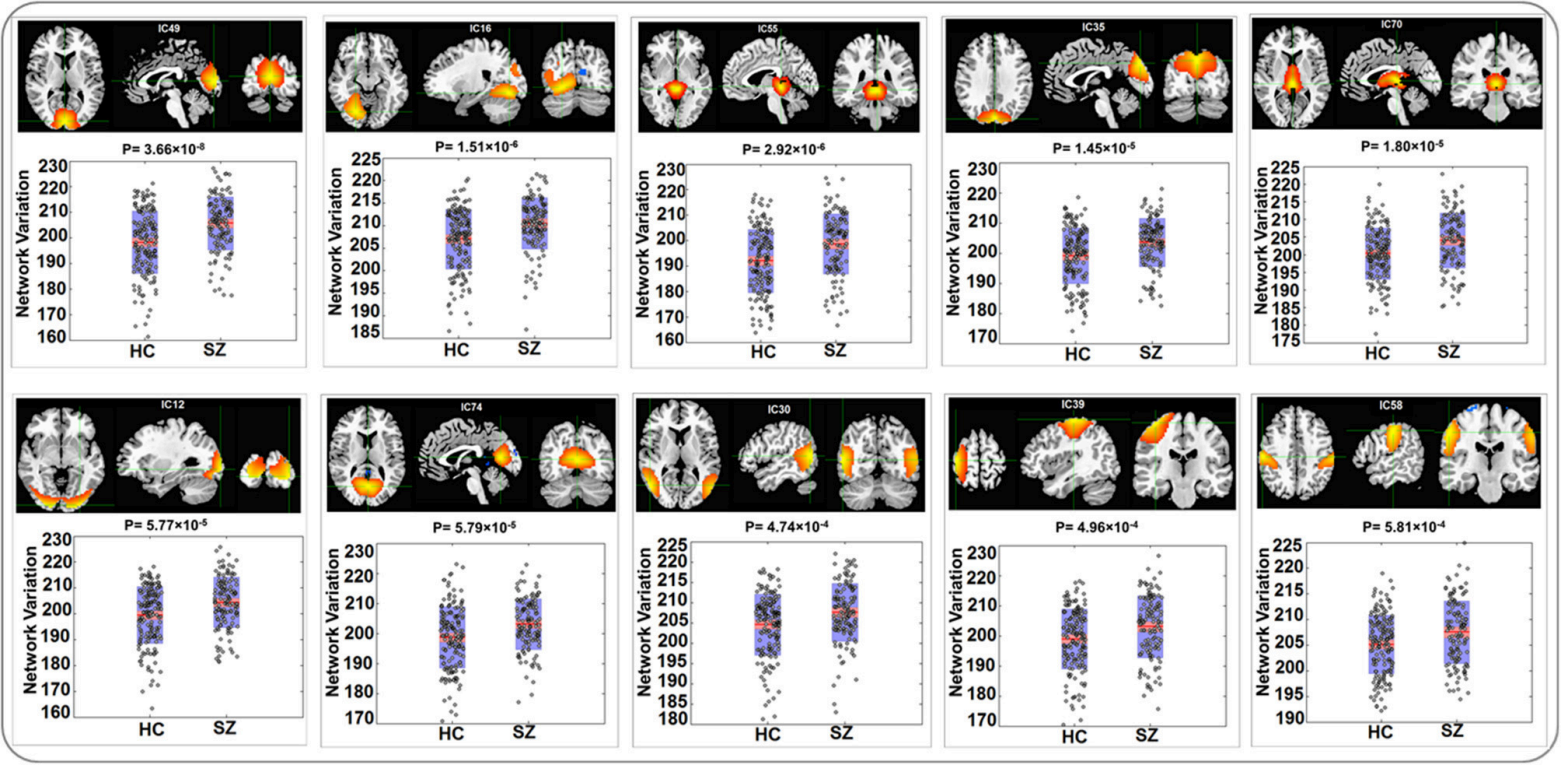
Spatial maps and boxplots of components showing significant group differences in RSN variability measured with Euclidean Distance.

**Figure 3:**
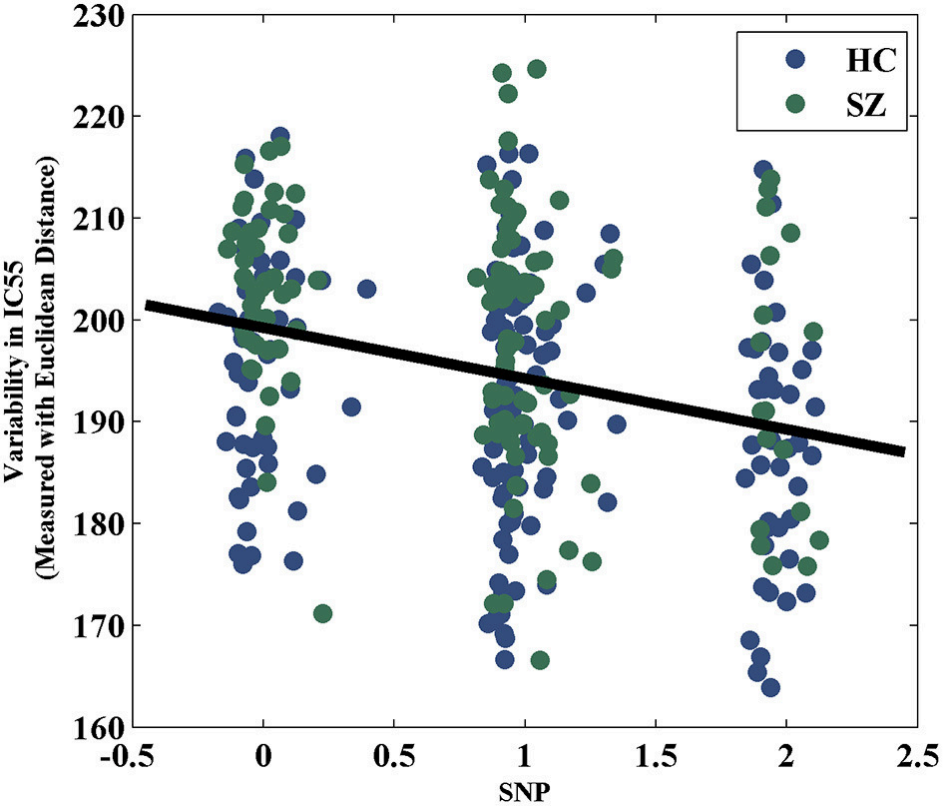
Scatterplot of rs11926768 and variability in IC55 measured with Euclidean distance.

The variability in RSN measured with Euclidean distance showed significant group differences (after Bonferroni correction) in 10 out of 75 components, majorly involved in visual and sensorimotor functions (IC12: inferior occipital gyrus; IC16: right fusiform gyrus; IC30: right middle temporal gyrus; IC35: left cuneus; IC39: right precentral gyrus; IC49: right cuneus; IC55: parahippocampal gyrus; IC58: postcentral gyrus; IC70: left thalamus and IC74: left lingual gyrus), as show in Figure 2. The directions of effects were consistent across these 10 components, with the patients presenting more deviation from the cohort-common networks compared with the controls. Variabilities in these 10 components all significantly and negatively correlated with global efficiency (mean correlation: −0.19, SD: 0.06). After controlling for diagnosis, 8 out of these 10 components still showed significant correlations with global efficiency. Meanwhile, patients presented significantly lower global efficiency (*p* = 9.89 × 10^−6^). For one of the group-discriminating components, IC55, a significant association (*t* = −4.99, *p* = 8.18 × 10^−7^, percentage of variance explained = 7.72%, passing Bonferroni correction) was observed between its variability measured with Euclidean distance and one SZ risk SNP, rs11926768 in *TRANK1*. Figure 3 shows the scatterplot of imaging genetic association between rs11926768 and IC55. No significant association was observed with CREB-BDNF SNPs or the PRS for SZ of either set.

When Pearson correlation was used to measure spatial variability, the same 10 components were noted to show significant group differences, which was not surprising given that features computed using Euclidean distance and Pearson correlation were highly negatively correlated (|r| > 0.99). The patient group presented lower correlations between the subject-level and group-level components, indicating more deviation from the cohort-common patterns. When measured with KL divergence (number of bins = 100), the variability in one of the cognitive control components (IC 26: Inferior Parietal Lobule) showed a significant group difference with patients showing higher divergence. For variability measured with either Pearson correlation or KL-divergence, no significant genetic association was noted.

### Variability in FNC

**Figure 4:**
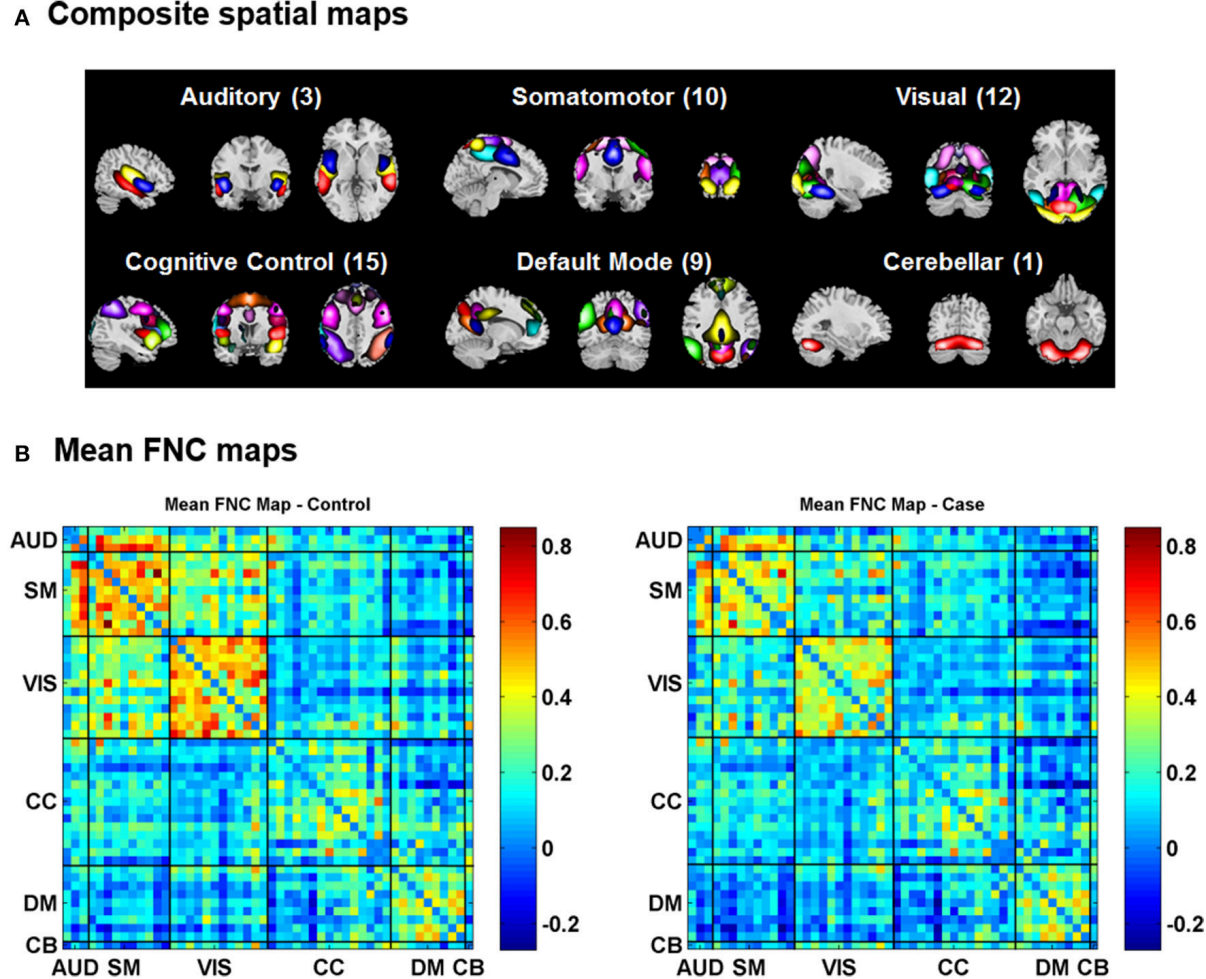
**(A)** Composite spatial maps of the resting-state networks. **(B)** Mean FNC maps of the control (left) and patient (right) groups.

**Figure 5:**
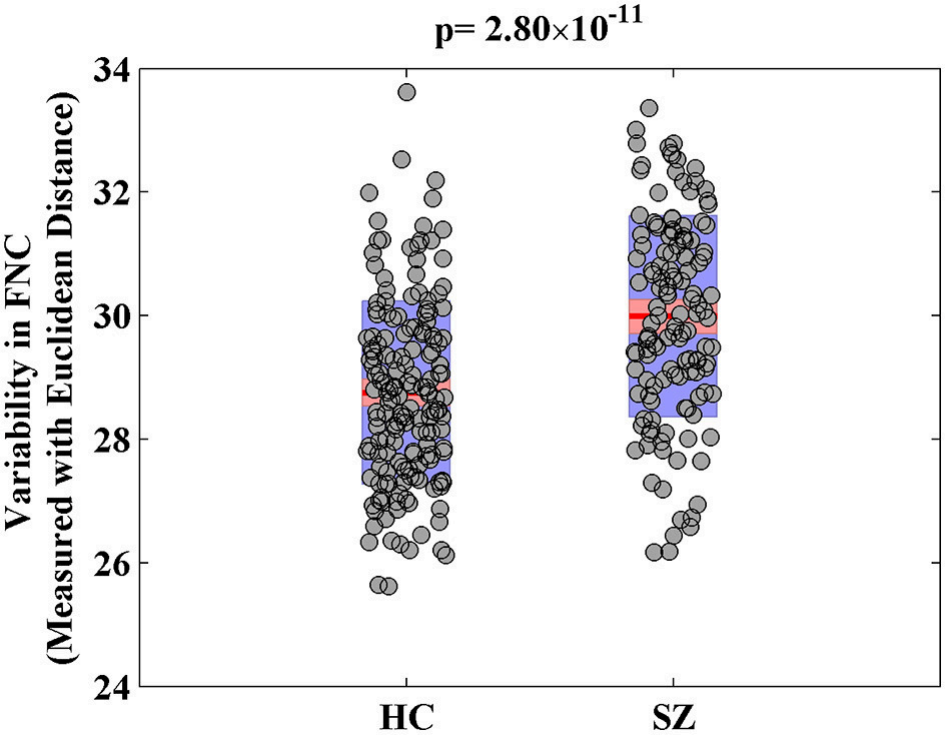
Boxplot of variability in FNC measured with Euclidean distance.

**Figure 6:**
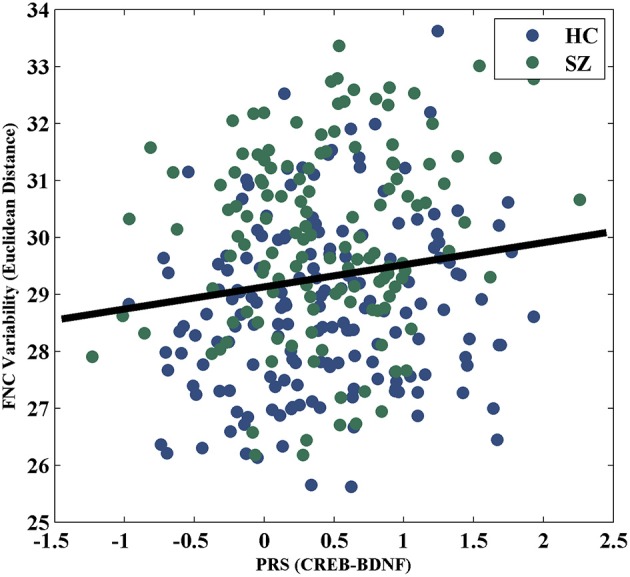
Scatterplot of PRS of CREB-BDNF SNPs and FNC variability measured with Euclidean distance.

For the visualization of FNC, The 50 selected RSNs were grouped into 6 functional domains, i.e., auditory (AUD), sensorimotor (SM), visual (VIS), cognitive control (CC), default-mode (DM) and cerebellum (CB), as show in Figure 4A. Figure 4B presents the mean FNC of the patient and control group, respectively. Significant group differences were noted when the variability in FNC was measured with Euclidean distance (*p* = 2.80 × 10^−11^) or Pearson correlation (*p* = 1.96 × 10^−11^). Given that these two features were again highly anti-correlated (*r* = −0.99), Figure 5 simply shows the boxplot of FNC variability measured with Euclidean distance for a demonstration. This group difference remained to be observed in the balanced subcohort of 134 patients and 134 controls, showing the same direction of change (*p* = 1.37 × 10^−8^ for Euclidean distance and *p* = 1.29 × 10^−8^ for Pearson correlation). The FNC variability significantly correlated with global efficiency: *r* = −0.28, *p* = 9.01 × 10^−7^ (Euclidean distance) and *r* = 0.28, *p* = 8.15 × 10^−7^ (Pearson Correlation), which remained to be significant after covarying out diagnosis (*r* = −0.21, *p* = 3.11 × 10^−4^ and *r* = 0.21, *p* = 2.97 × 10^−4^). Both FNC variabilities showed significant associations with PRS of the CREB-BDNF SNPs: *t* = 2.73, *p* = 6.64 × 10^−3^, percentage of variance explained = 2.41% (Euclidean distance) and *t* = −2.78, *p* = 5.83 × 10^−3^, percentage of variance explained = 2.48% (Pearson correlation). Figure 6 shows the scatterplot between PRS and variability in Euclidean distance for a demonstration. It can be seen that the higher the PRS for SZ, the larger the distance to the cohort-common pattern. Meanwhile, no significant association was noted with PRS of the PGC set or with individual candidate SNPs of either the PGC or the CREB-BDNF set.

## Discussion

We demonstrate that variabilities in RSN and FNC may serve as meaningful brain-based phenotypes in imaging genetic association analysis. We proposed to compute subject-specific variabilities in a group ICA-based framework and tested three different variability metrics. The results indicate that Euclidean distance and Pearson correlation are apparently more sensitive than KL divergence in terms of detecting group difference related to SZ. The identified group differences consistently point to SZ patients presenting increased deviation from the cohort-common network and connectivity pattern. Furthermore, the RSN and FNC variability were found to associate with individual SZ risk SNPs as well as cumulative polygenic risk score for SZ, suggesting a genetic regulation.

The identified 10 RSNs where variability measured with Euclidean distance or Pearson correlation showed significant group differences are mostly involved in visual and sensorimotor functions. Previous studies reported impaired functional activities in both visual and sensorimotor regions in SZ (Schröder et al., 1995; Butler et al., 2001, 2005). For instance, IC49 that showed the most significant group difference (Euclidean distance: *p*-value = 3.66 × 10^−8^; Pearson correlation: *p*-value = 3.39 × 10^−8^) highlighted the right cuneus region. This visual component has been found to show hyperactivation in SZ patients while performing facial emotion discrimination tasks (Seiferth et al., 2009; Habel et al., 2010), suggesting it plays an important role in dysfunctional emotion recognition in SZ patients. IC55 is one significant component that did not fall into visual or sensorimotor domain, highlighting the parahippocampal gyrus and thalamus. These regions have also been documented to show functional anomalies in SZ patients. Specifically, abnormal activity in parahippocampal gyrus has shown association with auditory hallucination in SZ (Silbersweig et al., 1995; Diederen et al., 2010; Escartí et al., 2010).

We also tested how variability in FNC differed between patients with SZ and healthy controls. The results concurred with the observations for variability in RSN, suggesting patients' FNC patterns showing larger distance and lower correlation to the mean FNC across the cohort. In the current analysis, we simply used the mean FNC as the cohort-common template for assessing subject-level variability. The fact that this may not be the ideal template does not hinder it being revealed that patients presented FNC patterns more distant from the cohort-common pattern than controls. The additional analysis using the subcohort of balanced patients and controls alleviated the possibility that the findings being biased due to the controls contributing more to the mean FNC. There have been extensive studies on FNC in the context of SZ, which has been documented for dysconnectivity in distributed brain regions including default-mode (Whitfield-Gabrieli and Ford, 2012), frontotemporal (Lawrie et al., 2002), and frontoparietal (Deserno et al., 2012) networks. While both hyper- and hypo-connectivity have been noted for individual networks in SZ, they appear to contribute in the same direction to the holistic variability feature, resulting in higher variability. Collectively, the analysis of variabilities in RSN and FNC suggest that the brains of patients with SZ deviate from the cohort-common pattern in both spatial and temporal domain.

The physiological interpretation of the variability features remains to be elucidated. For the variability in FNC, it likely captures the holistic network deviation from a “normal construction” in the time domain. We speculate this might underlie functional integration. To test this hypothesis, we assessed the correlation between variability in FNC and global efficiency, the latter suggested as a superior measure of integration (Achard and Bullmore, 2007; Rubinov and Sporns, 2010). As expected, we observed a significant negative correlation between variability in FNC and global efficiency, which indicated that more deviation from the mean FNC associated with lower global efficiency. This observation not only provides a physiological interpretation of part of the variance captured by the variability in FNC, it also lends support for the mean FNC to some extent reflecting a “normal construction” which likely relates to optimal global efficiency. The variability in RSN depicts the deviation of network's spatial construction from the cohort-common pattern. This feature also correlated with global efficiency, though less significantly than variability in FNC, which is not surprising given that global efficiency is largely a measure of FNC. Considering that variability in RSN indicates shift of network centers, we speculate this feature may directly relate to wiring cost, i.e., the anatomical distance between functional nodes (Alexander-Bloch et al., 2013), which awaits further verification.

Some preliminary genetic associations have been noted with the proposed variability features. The variability in IC55 showed a significant correlation with rs11926768 residing in *TRANK1*. As shown in Figure 2, IC55 highlights the parahippocampal gyrus and thalamus. *TRANK1* has been identified as one of the top risk genes for BD in a large GWAS (Chen et al., 2013a). Particularly, valproic acid, a treatment of mania, has been demonstrated to increase *TRANK1* mRNA expression, suggesting a relevance of this gene to emotional instability (Chen et al., 2013a). Meanwhile, *TRANK1* also confers SZ risk in PGC SZ GWAS (Ripke et al., 2014) and mood disturbances are common in SZ (Craddock et al., 2009). Together, these observations indicate a possibility of *TRANK1* being a common risk gene for SZ and BD and these two disorders partially converging on mood dimension (Ruderfer et al., 2014), for which some preliminary evidence has been provided (Goes et al., 2012). Echoing the genetic interpretation, the brain regions highlighted by IC55, parahippocampal gyrus and thalamus, are well characterized for their role in mood and emotion (Dasari et al., 1999; Strakowski et al., 2005; Drevets et al., 2008), lending support for the imaging genetic association in the current work. Another imaging genetic association was observed between PRS for SZ of CREB-BDNF SNPs and the variability in FNC. *BDNF*, regulated by *CREB* (Lonze and Ginty, 2002; Carlezon et al., 2005), plays an important role in synaptic plasticity (Schinder and Poo, 2000; Bramham and Messaoudi, 2005) which relates to functional connectivity (Sporns et al., 2000, 2005; Bullmore and Sporns, 2009; Van Dijk et al., 2010). Thus the observed association indicates a portion of SZ risk may disrupt synaptic plasticity which further leads to FNC deficits in SZ. Both imaging genetic associations warrant independent replications.

In summary, we propose to investigate subject-level variability from a cohort-common pattern in RSN and FNC as potential features for fusion of genetic and first-level resting fMRI data. We demonstrate that the proposed features have clinical relevance, revealing that patients with SZ present larger deviations from the cohort-common pattern in both spatial (RSN) and temporal (FNC) domain, and these variabilities show relations to global efficiency of brain connectivity. We also provide some preliminary evidence for genetic associations with the proposed features, lending support for their potential application to imaging genetic association. While the current findings answer a few top questions about the proposed model, one limitation lies in that we simply used a cohort-common pattern as the reference to compute the subject-specific variability. Our future work involves a comprehensive investigation on other potential references. We also expect to characterize the heritability of the variability features as well as complete the physiological interpretation before implementing the feature into the current parallel ICA framework.

## Ethics statement

This study was carried out in accordance with the recommendations of the Internal Review Boards of the participating institutions with written informed consent from all subjects. All subjects gave written informed consent in accordance with the Declaration of Helsinki. The protocol was approved by the Internal Review Boards of the participating institutions.

## Author contributions

JC and VC designed research; JC conducted analyses; JC and BR wrote the paper. The remaining authors contributed to the recruitment, data collection or processing for the participating cohorts of the study. All authors critically reviewed content and approved final version for publication.

### Conflict of interest statement

The authors declare that the research was conducted in the absence of any commercial or financial relationships that could be construed as a potential conflict of interest.
